# Numerical investigation of shipping noise in the Red Sea

**DOI:** 10.1038/s41598-024-56523-2

**Published:** 2024-03-11

**Authors:** Rihab Larayedh, Bruce D. Cornuelle, George Krokos, Ibrahim Hoteit

**Affiliations:** 1 Computer, Electrical and Mathematical Sciences and Engineering Division, King Abdullah University of Science and Technology, Thuwal, 23955 Saudi Arabia; 2 Scripps Institution of Oceanography, University of California San Diego, La Jolla, CA 92093 USA; 3 Institute of Oceanography, Hellenic Centre for Marine Research, 19013 Anavyssos, Greece; 4 Physical Science and Engineering Division, King Abdullah University of Science and Technology, Thuwal, 23955 Saudi Arabia

**Keywords:** Underwater shipping noise, Propagation modelling, Red Sea, Physical oceanography, Computational science, Environmental impact

## Abstract

Underwater noise pollution is a significant environmental issue that can have detrimental effects on marine ecosystems. One of the main sources of underwater noise pollution is ship traffic, which has been shown to negatively impact marine animals by masking communication signals and altering their behaviors. This study represents the first comprehensive analysis of underwater ship noise in the Red Sea, wherein noise maps of ships sailing through the main shipping lane in the Red Sea were simulated by integrating both anthropogenic and environmental variables. These maps offer valuable insights for policymakers, enabling them to make informed decisions and implement targeted mitigation efforts.

## Introduction

Underwater environments are considered as tranquil and silent habitats, but are in reality teeming with natural and biological sources of sound^1^. These include various weather events, such as thunderstorms and underwater earthquakes, and sounds produced by marine life for communication (e.g. whale and dolphin vocalizations, fish snapping or popping sounds, and crustacean stridulation). Many sea creatures rely on underwater sounds for communication and assessment of the surrounding environment, because, sound waves travel approximately five times faster than in air^2^, and vision is very limited due to optical absorption and scattering phenomena.

Increasing human activity in the ocean, including seismic exploration for oil and gas reserves, SONAR application for military exercises, commercial shipping, construction of offshore wind turbines, and sailing of cruise ships and leisure boats, affect underwater natural sounds. Since the onset of the industrial age, man-made ocean noise has continued to increase^3,4^. Although ships may not produce the loudest sound in the oceans, they are currently considered the most dominant and primary source of noise because of their ever increasing number. According to Statista, the global fleet of merchant ships engaged in international trade was   58,000 in 2022. Moreover, they emit signals over a wide frequency range of up to 10 kHz, but their traffic noise dominates below 500 Hz in the oceans^3,5^.

Underwater anthropogenic noise is thus recognized as a significant issue around the world and has detrimental effects on marine ecosystems^6^. Therefore, it must be mapped and monitored to identify areas with highest noise levels (NLs) and support decision makers to take necessary actions to reduce the impact of noise pollution on marine life. Several organizations and initiatives have already been initiated to address this issue. International Maritime Organization, the United Nations (UN) agency responsible for regulating global shipping, issued advisory guidance in 2014, specifically targeting noise emissions from commercial shipping vessels^7^. Various intergovernmental bodies such as the European Union^8^ and UN^9^ have taken proactive measures to address underwater noise pollution at a policy level. Additionally, various techniques, including acoustic monitoring, vessel tracking, and underwater noise modeling, have been proposed by researchers to map underwater ship noise. To this end, studies were conducted in the Eastern Mediterranean^10^, Northeast Atlantic^11^, and Slovenian Sea region^12^. In other countries, such as the USA, proactive measures have been employed to mitigate such issues. For instance, a study on underwater noise mitigation in the Santa Barbara Channel showcased dedicated efforts in this regard^13^.

Shipping-noise mapping and its integration into environmental impact assessments is well-established globally, and extensive monitoring studies are underway in several countries. Viola et al.^14^, Cafaro et al.^15^, and Pieretti et al.^16^ have considerably advanced the understanding of shipping-related noise in Italy. Rako et al.^17^, Picciulin et al.^18^, and Vukadin^19^ studied the impact of ship traffic in Croatia (Northern Adriatic Sea). Širovic’ and Holcer^20^ explored maritime NLs in Montenegro, and researchers from Greece^21^, Slovenia^12,22^, and Australia^23^ (the Great Barrier Reef) have focused on underwater noise. We focused on the Red Sea, a highly distinctive basin region characterized by unique physical patterns, particularly realted to temperature and salinity. The Red Sea is one of the busiest waterways in the world; therefore, investigating the ship noise in this ecologically significant area is complex.

The Red Sea is a unique marine environment and a home to houses diverse marine life. It hosts one of the longest continuous living coral reefs in the world, supporting an ecosystem involving around 350 different coral species^24,25^. More than 800 ships sail through the Red Sea every day, the majority of which are large vessels, including crude oil tankers and cargo ships. Despite the importance of coral reefs therein, the potential risks of such high ship traffic on its ecosystems have not yet been investigated in depth. Only a few studies have investigated the impact of underwater noise pollution on species in specific areas in the Red Sea. The impact of underwater noise on dolphins in the Gulf of Aqaba and some specific species (Lambis and Tridacna maxima) of coral reefs in the northern Red Sea has been investigated^26,27^. However, the spatial and temporal distributions of underwater noise in the Red Sea have not yet been investigated.

Herein, the maps of noise generated from ships along the main shipping lane in the Red Sea are generated to understand the spatiotemporal distribution of underwater shipping noise and its seasonal variation. The discipline of modeling underwater sound propagation has a long history, primarily originating from its military applications using SONAR technology. Over the years, various modeling approaches have been developed, each possessing distinct suitability based on factors such as acoustic frequency range, water depth, computational requirements, and environmental spatial variability^2^. Among these, the parabolic equation method was used herein for low-frequency predictions and complex bathymetry in shallow and deep waters^28^. However, noise map generation was computationally demanding due to significant number of noise sources and large computational domains (1 snapshot required   4480 CPU hours and two snapshots were computed per day for 2 months, resulting in a total of 124 snapshots, which amounted to 560,000 core hours). The underlying codes were optimized and the computational power of KAUST’s supercomputer, Shaheen, was leveraged to generate high-resolution maps and manage the substantial computational costs associated with the endeavor. We used an acoustic propagation model, the range-dependent acoustic model^29–31^, which simulates the propagation of noise from ships based on inputs of shipping traffic data, and underlying ocean conditions. These results will allow us to comprehensively understand underwater noise pollution in the Red Sea and detect areas with the highest risk for developing effective countermeasures and management strategies.

## Methods

Numerous parameters have to be considered for underwater sound modeling, which vary with location, time, and environmental conditions such as sound speed, density, ship positions, bathymetry structure, and attenuation^32^.Therefore, these parameters have to be continually updated for developing a representative and effective underwater sound model.

### AIS data and source modeling

**Figure 1:**
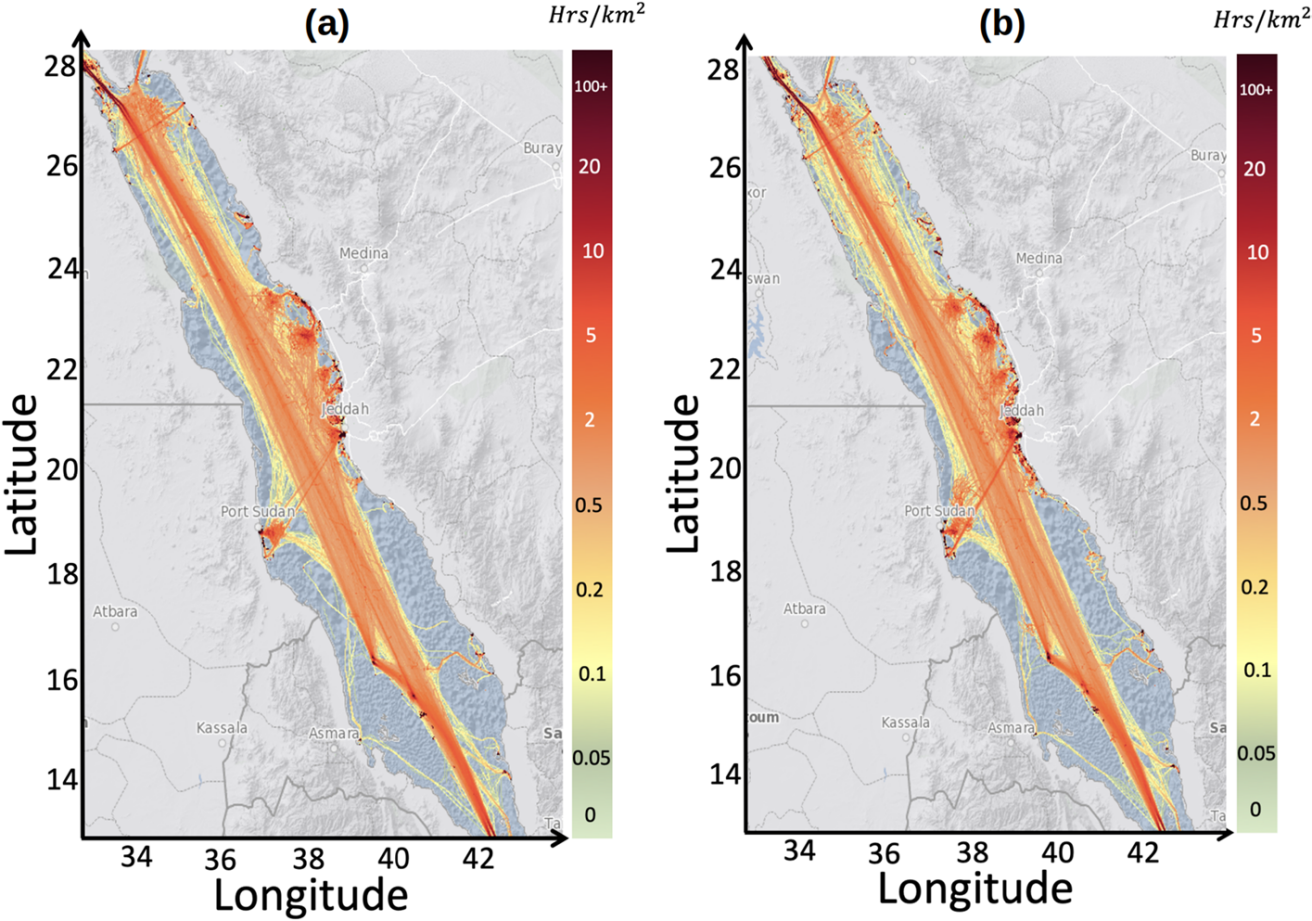
A map of Red Sea shipping traffic density as a fraction of total time per square km (color) for (**a**) January 2021 and (**b**) July 2021^33^. Map produced using Matlab R2023a (https://www.mathworks.com).

AIS, a VHF-based system, facilitates message exchange among ships; AIS is mandatory for ships over 300 tons to enhance navigation and safety. Vessel positions in the Red Sea were derived from AIS data for the area with latitudes and longitudes of 10^∘^–30^∘^ and 30^∘^–50^∘^, respectively, for 2 months (January and July) in 2021. Vessel density maps for the Red Sea based on historical AIS data for January and July 2021 are shown in Fig. 1 using the data provided by Global Maritime Traffic Density Service^33^. Herein, only some main vessel types were categorized, namely tankers, bulkers, container ships, open hatch vessels, and vehicle carriers.

These ships were chosen due to their dominance in the area in January and July. For example, tankers accounted for 25% of the total ship traffic. Thus, the Red Sea is a vital shipping route for tankers, particularly those involved in transporting oil products. Additionally, different carriers constituted 15% of the total ship traffic, whereas bulkers and open hatch ships collectively represented 13% of the maritime activity. Container ships constituted 9.3% of the total ship traffic, underscoring the role of the Red Sea in facilitating the global movement of goods through containerized shipping. In contrast, passenger ships constituted only 0.4% of the total ship traffic, whereas fishing vessels, including fishery patrol vessels and fishery vessels, represented   3% of the total ship traffic. These fishing vessels primarily operate near shore.

A moving ship produces noise at various frequencies and levels from propeller cavitation, engine and hull vibrations, and bow wave. Among these, propellers are the main source of noise^34^. The features of the noise generated depend on factors such as the ship’s type, design, construction, maintenance status, and navigation conditions (e.g., speed and load). The sound produced in a specific direction can be characterized by the spectral density of the source level (SL), which is a function of frequency (known as the acoustic signature) and is measured in dB re 1 $\upmu$ Pa^2^ m^2^/Hz.

SLs for ships must be accurately determined to predict sound propagation in the marine environment as errors in SL can impact the acoustic field. However, this process is expensive and complex; therefore, approximations are commonly used. Herein, we used a spectrum model, JOMOPANS-ECHO^35^, developed in response to specific requirements for marine acoustic mapping. It addresses some limitations of pre-existing models such as RANDI1.3^35^ and WH02^35^

1 $$\begin{aligned} L_{s_f \mathrm {~J}-\textrm{E}}(f, V, l, C)=L_{S_{f, 0}}(f, C)+60 \log _{10}\left( V / V_C\right) \textrm{dB}+20 \log _{10}\left( l / l_0\right) \textrm{dB}. \end{aligned}$$

Equation (1) expresses the source spectral density level, denoted as $L_{s_f \mathrm {~J}-\textrm{E}}(f, V, l, C)$, containing variables such as frequency ($f$), ship speed ($V$), ship length ($l$), vessel class ($C$), and reference length ($l_0=91.44\,\text {m}$). The reference spectrum per vessel $L_{S_{f, 0}}(f, C)$, is given as follow^35^:

2 $$\begin{aligned} L_{S_f, 0}(\widehat{f}, \textrm{C})=K-20 \log _{10}\left( \widehat{f}_1\right) \textrm{dB}-10 \log _{10}\left( \left( 1-\frac{\widehat{f}}{\widehat{f}_1}\right) ^2+D^2\right) \textrm{dB}, \end{aligned}$$

where $K=191$ dB and $D=3$ ( note that $D_{\text {cruise vessel}} = 4$) . The variables include normalized frequency ($\widehat{f}=f/f_{\text {ref}}$) and normalized frequency based on reference speed ($\widehat{f}_1=480 \ \text {Hz} \times (V_{\text {ref}}/V_C$)), where reference frequency is 1 Hz and reference speed is equal to 1 kn. As large vessels such as container ships, vehicle carriers, bulkers, and tankers are targeted herein at frequencies below 100 Hz, an additional peak is introduced^35^:

3 $$\begin{aligned} L_{\textrm{S}_f, 0}(\widehat{f}<100, \text{ Cargo } )=K^{L F}-40 \log _{10}\left( \widehat{f}_1^{L F}\right) \textrm{dB}+10 \log _{10}(\widehat{f}) \textrm{dB}-10 \log _{10}\left( \left( 1-\left( \frac{\widehat{f}}{\widehat{f}_1}\right) ^2\right) ^2+\left( D^{L F}\right) ^2\right) \textrm{dB}, \end{aligned}$$

where $K^{LF} = 208$ dB and $D^{LF} = 0.8$ for container ships and bulkers and $D^{LF}= 1$ for vehicle carriers and tankers. The normalized frequency based on reference speed ($\widehat{f}_1^{LF}=600 \,\text {Hz} \times (V_{\text {ref}}/V_C)$) is also introduced. The SL in the decidecade frequency bands is converted by adding $10 \log _{10}(0.231 \widehat{f}) \,\textrm{dB}$.

The ship types considered herein were further categorized into four main types for in-depth analysis, as shown in Table 1:

**Table 1:** Characteristics for each ship category with effective ship length subdivisions.

Categories	Types of ship	$V_{c}$^35^ (kn)	Ship length (m) per range (effective %)	Effective ship speed (kn)
C1	Tankers	12.4	100–200: 163.38, (43.5% ) 200–300: 251.92 (41%) > 300: 330.6 (11.3%)	12
C2	Bulkers and open hatch vessels	13.9	100–200: 166.03 (43%) 200–300: 223.2 (36.5%) > 300: 306.03 (9.33%)	11.1
C3	Container ships	18	100–200: 170.85 (39.4%) 200–300: 239.54 (30%) > 300: 356.41 (18.6%)	13.8
C4	Vehicle carriers	15.8	100–200: 189.36, (42.8%) 200–300: 279.79 (39.6%) > 300: 322.12 (15%)	13.5

**Figure 2:**
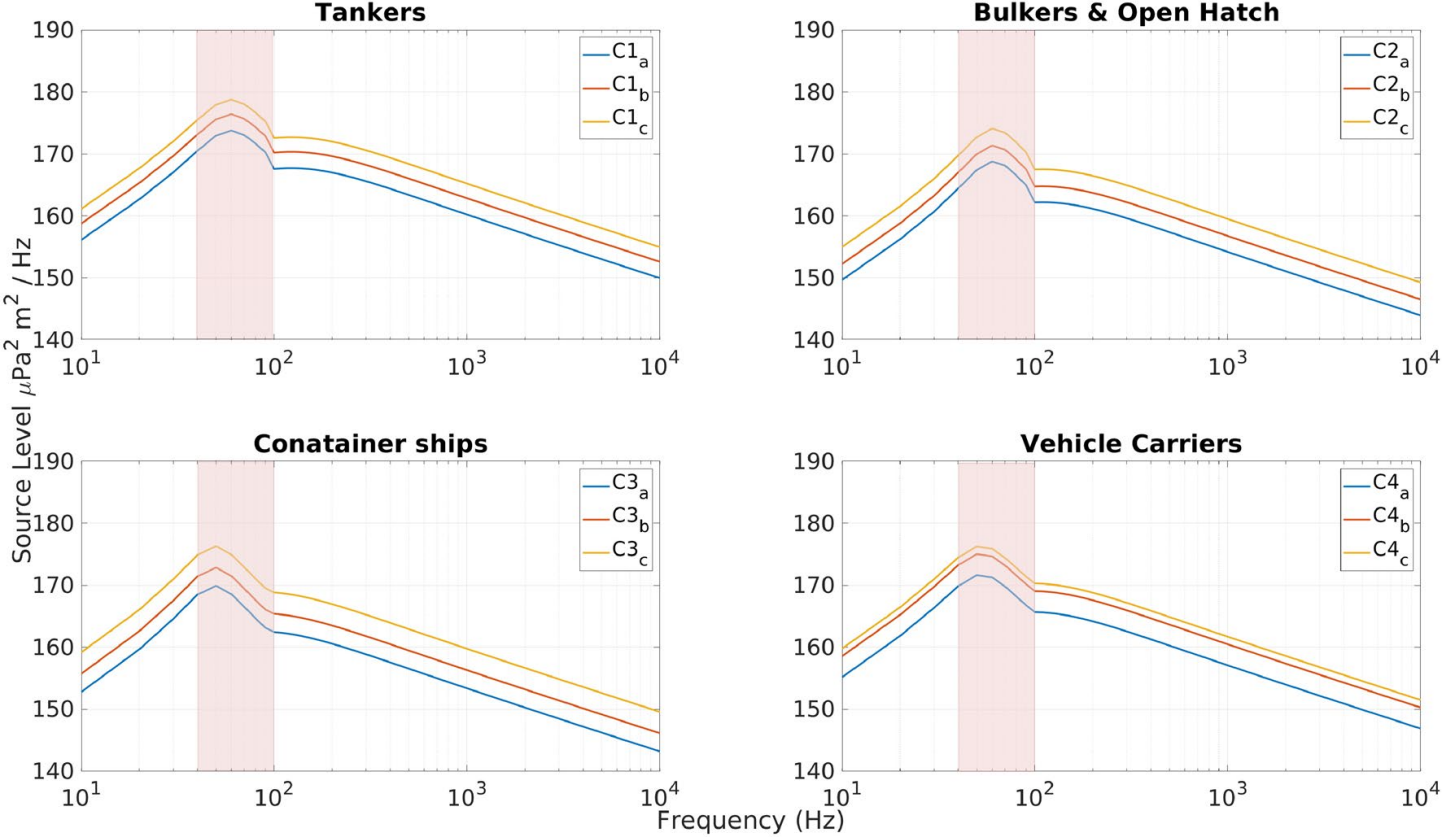
Predicted source levels using data from Table 1 for four main ship categories: (C1) tankers, (C2) container ships, (C3) vehicle carriers, and (C4) bulkers. The ship categories are further delineated by length ranges: (**a**) 100–200 m, (**b**) 200–300 m, and (**c**) > 300 m, each representing a distinct subset within the specified category. The propagation calculations are constrained to the highlighted frequency band (40–100 Hz),where the expected mix of ships exhibits the highest power.

The ship characteristics (Table 1) are input to the reference spectrum model used for estimating the SLs of marine shipping (Fig. 2), as detailed in an article by MacGillivray and de Jong^35^. The reference speed ($V_c$) is adopted from their study, with a source depth of 6 meters. In addition, effective ship speed was calculated by averaging the speeds of moving vessels in each category observed in the Red Sea during January and July. The effective ship length was categorized into three ranges: 100–200, 200–300, and >300 m. Vessels with lengths of < 100 m were eliminated because only 7.5% of ships in the dataset were < 100 m long.

### Environmental parameters

**Figure 3:**
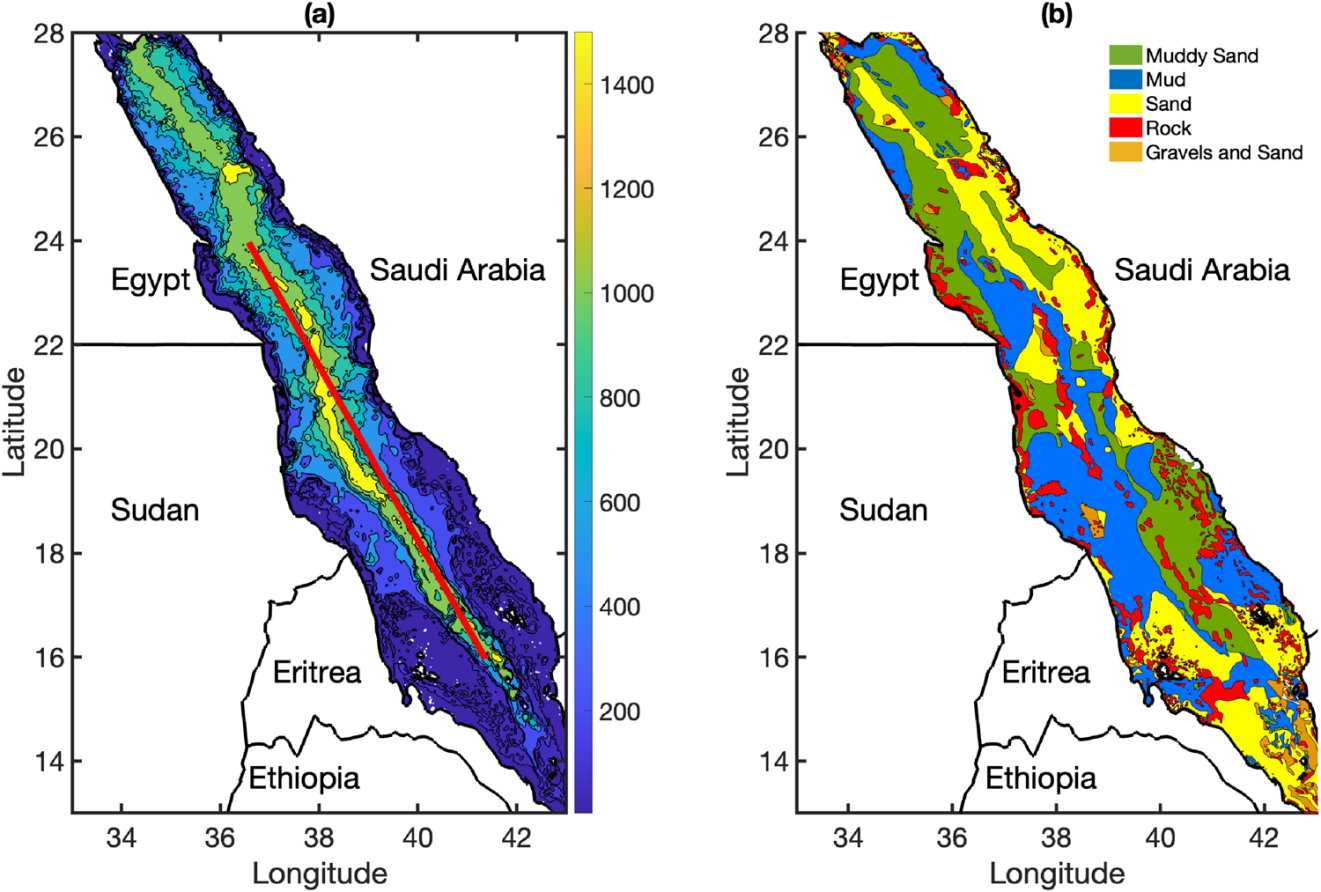
(**a**) Bathymetric data used in this study, where colors show the depth in meters. The red line indicates the section corresponding to the subsequent figures (**b**) Seafloor sediment characteristics (colors show sediment type). Map was produced using Matlab R2023a (https://www.mathworks.com).

Sea bottom topography is crucial for sound wave propagation. The interaction of acoustic waves with the seafloor includes wave scattering, transmission, and reflection. The bathymetry data used for the modeling are shown in Fig. 3a, which were obtained from the General Bathymetric Chart of the Oceans digital atlas in 30-arc-second intervals. The data show that the Red Sea is not an ultradeep basin and comprises 45% shallow waters (< 300 m), 52% mid-waters (300–1500 m), and only 3% deep waters (> 1500 m) (Fig. 3a).

The propagation of sound waves is influenced by ocean depth, topography, and sediment characteristics. To accurately integrate these data into the model, reliable data on seafloor properties must be obtained. However, obtaining such data can be challenging, particularly in basins such as the Red Sea with limited sedimentological information.

The sediment types in the area were determined from the geological and geomorphological maps from the scientific literature to estimate the relevant coefficients for sound propagation models. The modeling parameters, such as sound speed ($C_{b}$ in m/s), density in sediments ($\rho _{b}$ in g/cc), and attenuation in sediments ($\alpha$ in dB/wavelength), were determined based on the fundamental principles of geoacoustic properties^36–39^. These references describe the fundamental principles, measurement techniques, and practical examples of seafloor materials across various oceanic environments, providing a comprehensive overview of seafloor acoustic properties. The geoacoustic properties were based on a map produced by the French Hydrographic and Oceanographic Service^40^. Figure 3b shows that mud and sand accounted for 82% of the seafloor composition, whereas rock and other sediments (e.g., silt and gravel) accounted for 14% and 4%, respectively. The seafloor model used herein was a semi-infinite, homogeneous medium without shear, consistent with the geological and geomorphological information obtained from the existing maps.

Coming to the sound speed computations, there are multiple formulas to calculate sound speed and such formulas may be found in oceanographic textbooks. Here, we adopted the Del Grosso-Mackenzie formula^41^. The sound speed profiles in the Red Sea were obtained from the daily averaged profiles of temperature and salinity using a high-resolution (1 km) general oceanic circulation model. The high-resolution dataset was simulated by a regionally tuned and extensively validated configuration of the MIT general circulation model (MITgcm)^42^, covering the entire Red Sea basin and the adjacent Gulfs, namely the Gulf of Suez, the Gulf of Aqaba, and the Gulf of Aden^43^. The model implemented with 50 vertical layers, the vertical resolution varied from 4 m at the surface to 300 m at the bottom. The model output was previously used for various studies on the Red Sea and successfully described the circulation dynamics ranging from large-scale overturning circulation^44,45^ to vigorous mesoscale eddy activity^46,47^. This model accurately reproduced the seasonality and structure of the upper mixed layers and the depth of the thermocline in the Red Sea^43,48^.

**Figure 4:**
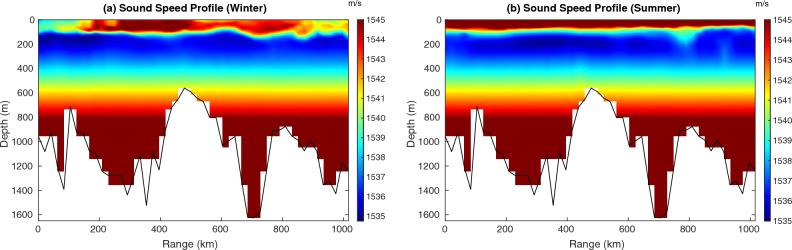
Spatial and temporal variability of sound speed profiles in the Red Sea, in meters per second (color), along the section highlighted in Fig. 3a, with the source and receiver located in the southern and northern Red Sea, respectively. The black line shows bottom topography (resolution: 5 km).

Figure 4 shows the spatial contrast in sound speed profiles spanning a distance of 1000 km across the Red Sea for 1 day in summer and 1 day in winter. These fluctuations in sound speed predominantly resulted from temperature variations. Figure 4a shows a surface duct due to minimum sound speed near the water surface. Figure 4b shows the sound speed profile in summer that does not show a near-surface duct. The black line shows bottom topography in both the plots.

### Acoustic field computation

Some parameters are set beforehand for computing acoustics based on the specific scenario being modeled. For example, the vertical resolution was set to 1 m herein for investigating the propagation of sound in the vertical dimension. The depth of the ocean floor and the number of sediment layers, which vary with depth, affect the propagation distance in the vertical plane. A distance of 1 km was chosen for the horizontal resolution based on the data obtained from the general oceanic circulation model of the Red Sea, which provides daily profiles for salinity and temperature with a resolution of 1 km. Additionally, the bathymetry data used for the analysis also had a resolution of 1 km. Each source had different transmission frequencies that must be taken into consideration in the model. Computations were performed in a narrow frequency band ranging from 40 to 100 Hz using 1-Hz bins. Computations were performed across 61 discrete frequencies.

To compute acoustic propagation, the input variables that need to be specified include the location of ships in the region and their specifications along with other relevant environmental factors and the locations of all receivers, where the sound pressure levels (SPLs) are calculated. The 2D $\times$ N method was used^49–51^ to compute the acoustic field. Owing to the high computing capacity provided by the KAUST supercomputer, Shaheen, a high azimuth angular resolution of 0.57^∘^ was used. Such a high angular resolution considerably enhances the resolution of the results, allowing for a more precise analysis of sound propagation dynamics in the region but at the expense of increased computation time and requirement of a powerful computing system.

Each source emits sound with a specific maximum propagation distance, $R_{max}$, a parameter often assumed in the context of the horizontal plane, referred to as the radius of the propagation disk. However, the maximum range is not a fixed constant but varies based on specific circumstances and the particular sources involved. Given that the majority of ships navigate through the main shipping lane of the Red Sea, a distance of 150 km or less is typically used. This limitation arises due to two primary reasons. First, beyond this distance, sound reaches the land as the Red Sea width is approximately 300 km. Second, other ships (sources) may be present within this range, further emphasizing the importance of considering the dominance of louder sounds in the surrounding maritime environment. Based on the environmental input the propagation loss (*PL*) information on sound attenuation was generated as the sound propagated through water.

For each frequency (*f*), the *SL* corresponding to each ship (*i*) is selected and combined with the *PL* to determine the spectral power at that frequency for each receiver range (r) by integrating the spectral density power as follows:

4 $$\begin{aligned} P_{\text {rms-spectral}}(i, r) = \sqrt{\sum _{f} F_{S}(f,i)* F_{P}(f,i,r)}, \end{aligned}$$

in accordance with the definitions outlined in ISO 18405, $F_{P}$ represents the propagation factor for PL, while $F_{S}$ represents the source factor relating to SL. After obtaining the spectral power at each frequency for all receiver positions, the total *NL* for each cell is calculated by adding the spectral powers of all ships that contribute to the NL at that cell. The concept of closest neighborhood is applied during the conversion from radial (r) to cell (*x*, *y*). Notably, when multiple points contribute to the acoustic information in a given cell the model takes the average of those points.

5 $$\begin{aligned} NL_{\text {rms-total}}(x, y) = 10 \times \log _{10}\left( \frac{\sqrt{\sum _{i} P_{\text {rms-spectral}}(i, r)^2}}{P_0}\right) \,\text {dB}, \, with \; p_0= 1\,\upmu \text {Pa}. \end{aligned}$$

## Results

### Seasonal distribution of ship noise propagation

**Figure 5:**
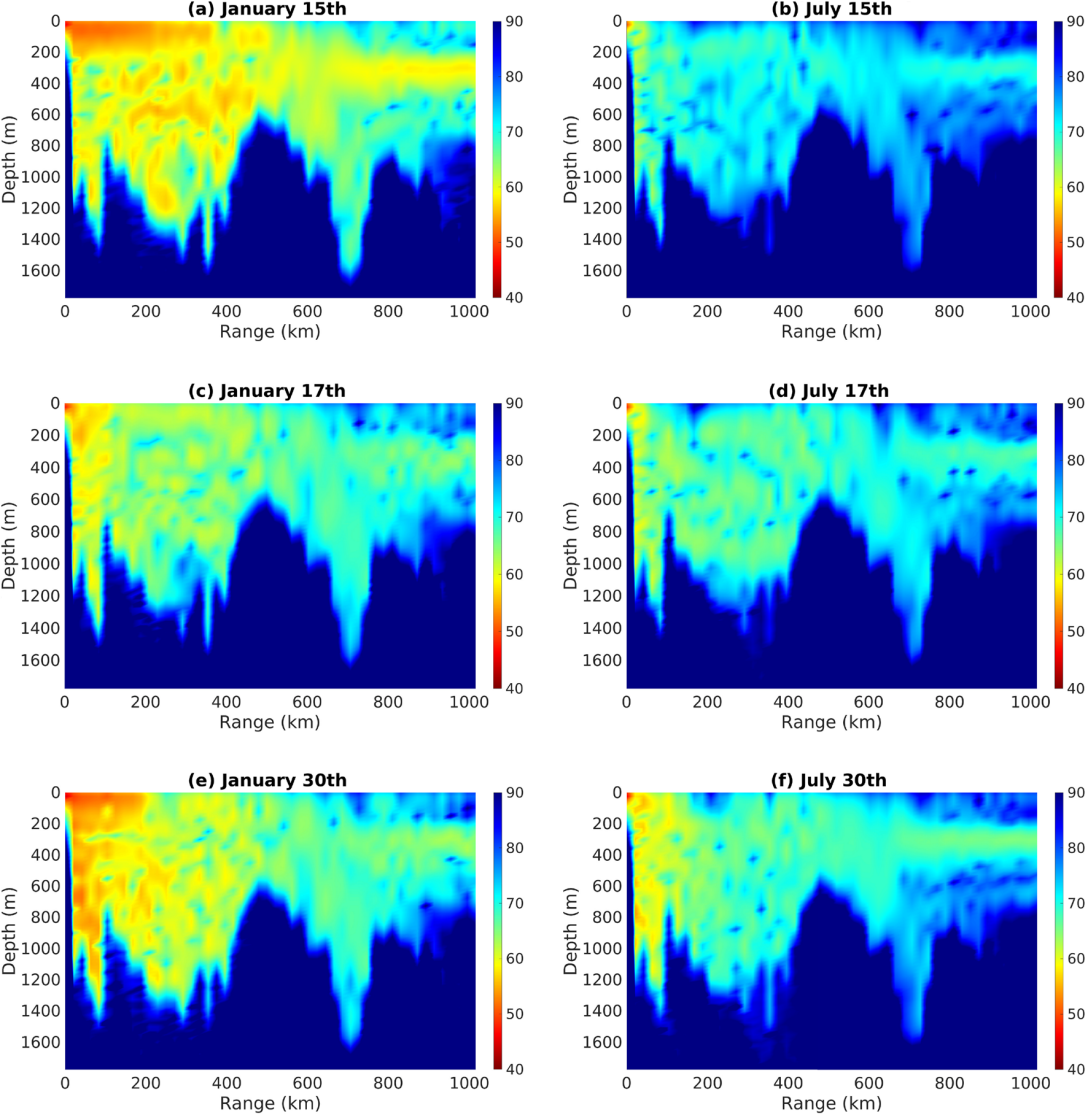
Daily propagation loss (PL) computed along the same path as Fig. 4 at a frequency of 90 Hz and source depth of 40 m from: (**a**) January 15th, (**b**) July 15th, (**c**) January 17th , (**d**) July 17th, (**e**) January 30th, and (**f**) July 30th. Colors indicate PL.

Figure 5 displays the PL computed at a frequency of 90 Hz along a path in the Red Sea, originating from a source depth of 40 m. Notably, the graph reveals a distinct seasonal trend in PL. In January (Fig. 5a,e), PL is particularly lower near the surface, indicating less acoustic attenuation, resulting in a PL of 50–60 dB at approximately 200 km (Fig. 5a). The surface duct allows the sound to be trapped in a region close to the surface before interacting with the bottom layer, resulting in the observed characteristics.

In contrast, the PL substantially increases near the surface in July (Fig. 5b,f), indicating higher attenuation along this identical section of the path. This difference is attributed to the varying sound speed profiles between January and July, corresponding to the winter and summer seasons respectively, particularly the presence of surface duct during winter. The downward-refracting sound speed profile typically results in a consistent energy loss into the bottom, except for instances wherein a duct is present near the surface. This possibly plays a vital role in explaining the variations between winter and summer, although these distinctions may not be particularly pronounced. Figure 5c,d show a small difference between months as the surface duct is less important.

### Spatial distribution of ship noise propagation

**Figure 6:**
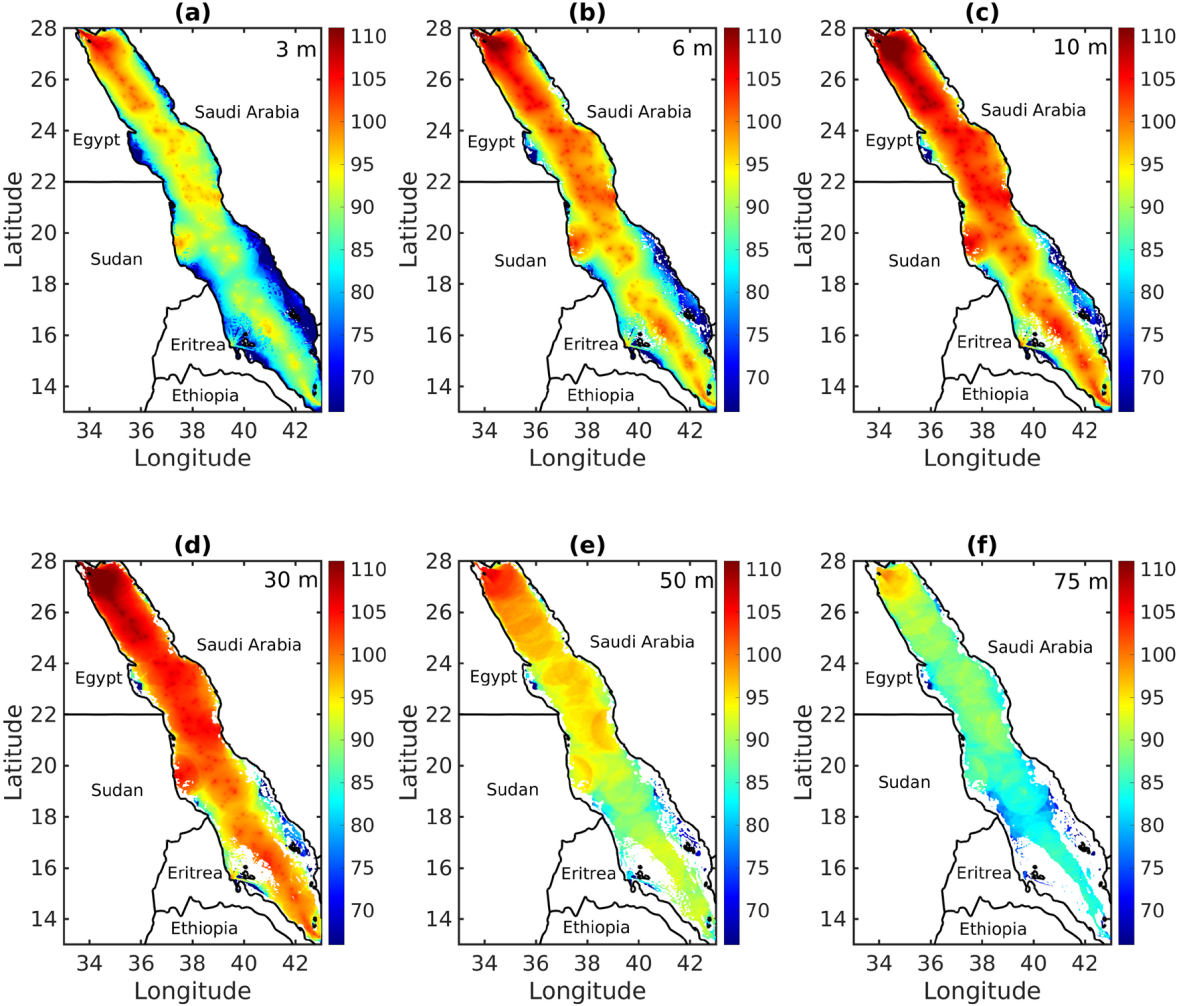
Predicted spectral noise level (*dB* *re* $1\,\upmu \,\text { Pa}^{2}$/Hz) distribution, represented by colors, on 11 January 2021 in the Red Sea averaged over the frequency band: 40–100 Hz at depths of (**a**) 3 m, (**b**) 6 m, (**c**) 10 m, (**d**) 30 m, (**e**) 50 m, and (**f**) 75 m. Map was produced using Matlab R2023a (https://www.mathworks.com).

To assess spatial variability, instantaneous ship noise maps were produced daily for January and July. Figure 6 shows the predicted spectral NL (*dB* *re* $1\,\upmu \text {Pa}^{2}$/Hz) distribution on January 11, 2021, at six depths. The maps show a peak in ship NLs in the northern part of the Red Sea at different depths, with the highest levels at 10 and 30 m (Fig. 6c,d) and the lowest levels at 75 m (Fig. 6f).

The observed patterns in the generated maps were consistently distinct. In Fig. 6, the sound intensity is relatively low near the surface, reaches its highest level at depths of 10–50 m, and decreases as the depth increases. Spatially, the SPL is notably higher within the main shipping channels, exceeding 120 dB, due to intense maritime activities. Moreover, unevenly distributed NLs were observed in the Red Sea due to a combination of factors. First, bathymetric features, such as extensive shallow banks, numerous islands, and coral reefs, acted as barriers that impeded sound propagation and created shadows, thereby protecting the coasts from noise generated by ships. Additionally, the limited routes for ship traffic, which is concentrated in the main shipping lane, contribute to lower NLs in the southern region. Conversely, the northern side of the shipping channels experiences elevated NLs primarily due to bathymetry and the accessibility of ports, leading to increased vessel activity and noise.

The phenomenon of elevated NLs in shallow water areas, such as those situated near the Suez Canal or the Sudan port, is an interesting subject. These regions, due to their proximity to major shipping routes, experience high levels of noise. The Suez Canal known for its strategic significance as a vital maritime passage connecting the Mediterranean Sea and Red Sea, attracts a substantial amount of shipping traffic. A separate study focusing on the Eastern Mediterranean region further supports this observation, substantiating the presence of high NLs in the shallow region of the Suez Canal^10^. An additional figure has been added to the Supplementary Material (Fig. S2), featuring the sound pressure level for the 63 Hz decade band. This addition aligns with the prescribed indicator for continuous underwater sound as delineated in the EU Marine Strategy Framework Directive.

### Noise level exceedance

**Figure 7:**
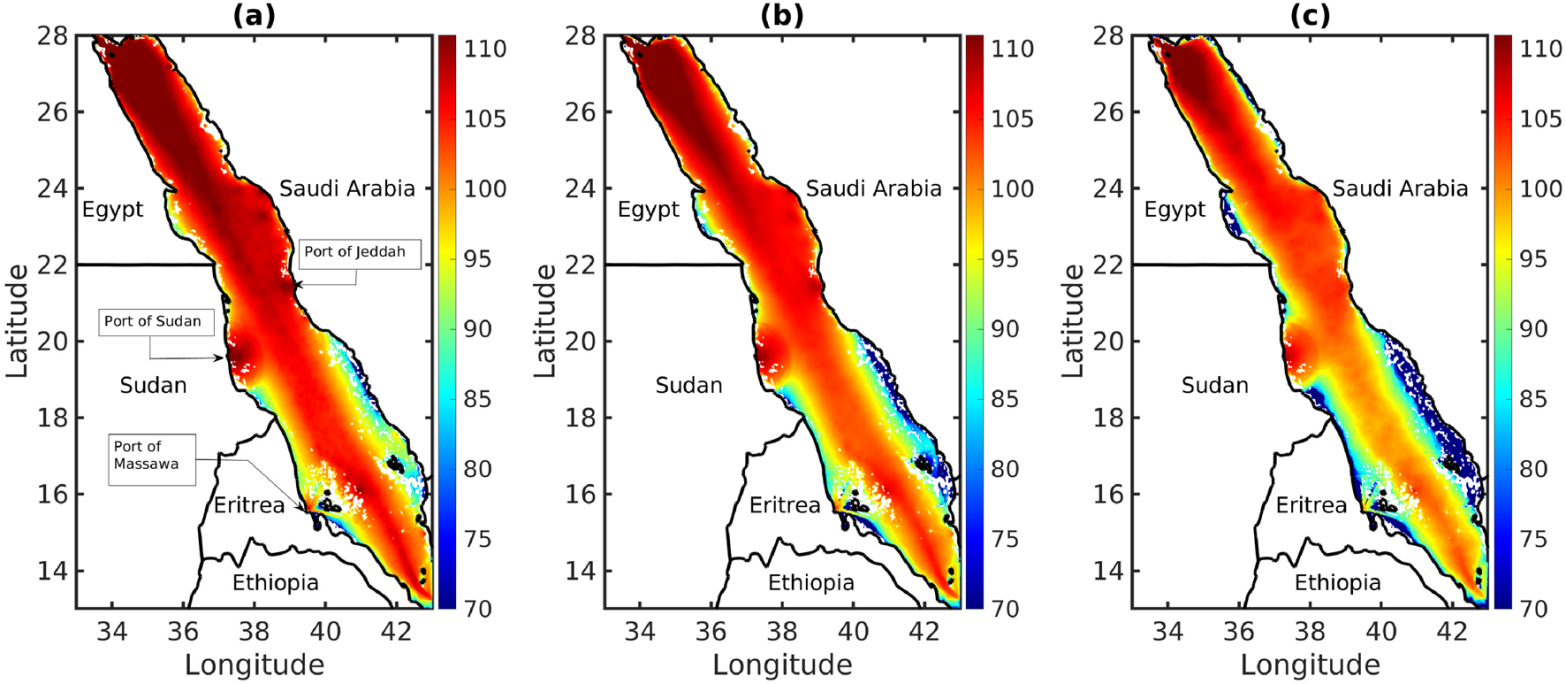
Median predicted spectral noise level (*dB* *re* $1\,\upmu \, \text {Pa}^{2}$/Hz) color distributions in the Red Sea at the depth of 12 m, averaged over the frequency band (40–100 Hz) for 3 quantities over the months January and July, 2021. (**a**) $L_{10}$, (**b**) $L_{50}$, and (**c**) $L_{90}$. Map produced using Matlab R2023a (https://www.mathworks.com).

The NL exceedance, which indicates how often a specific sound pressure level is exceeded, is valuable for understanding the persistent extreme NLs experienced by marine organisms. It can be represented as a percentile of an SPL time-series, where a certain value is surpassed by a certain percentage of observations. Figure 7 shows the NL exceedance for the entire combined duration of January and July, with two daily observations for each of the 2 months. The figure shows results for both months because the modeled results exhibited very similar graphical information for both months. The SPL metrics namely $L_{10}$, $L_{50}$, and $L_{90}$, provide insights into the probability of sound intensity in noise environments. $L_{10}$ indicates that noise exceeds this threshold for 10% of the time. Conversely, $L_{50}$ represents the level surpassed for 50% of the time, making it the statistical midpoint of noise readings and reflecting the central tendency of noise distribution. In contrast, $L_{90}$ represents the level exceeded for 90% of the time, indicating that noise is above this threshold for the majority of cases. This relationship illustrates the progression of SPLs from baseline levels ($L_{90}$) to more intermittent occurrences ($L_{50}$) and further to extreme events ($L_{10}$) based on the modeling of ship noise events. In the southern regions, underwater topography plays a crucial role in reducing noise and prevents the noise from reaching coral reefs edges. Conversely, NLs are considerably higher in and around port areas. A significant distinction between the density maps in Figs. 1 and 7 is the representation of sound propagation.

While the information about the validation and calibration of the developed mapping model is limited, these noise maps present a preliminary resource for tracking trends in shipping NLs. A comparative analysis of the existing studies reveals compelling patterns^10,12,52^. Notably, a correlation exists between ship density and sound distribution on the map, with high concentrations observed in areas with high maritime traffic. The map further shows that sound attenuates as it approaches coastlines. Compared with northeast Atlantic^11^, where ships tend to hug the coasts, the Red Sea area is unique because shipping lanes traverse in this narrow expanse, resulting in a distinct soundscape. The narrow geography of the Red Sea, which is approximately 300-km wide allows sound to reach the coastlines as ships navigate through its central axis. Despite lacking formal validation, these consistent observations, coupled with the unique characteristics of the Red Sea, strengthen the tool’s utility for preliminary assessments and managerial insights.

## Discussion

Herein, the impact of ship traffic on underwater noise pollution in the Red Sea is investigated by generating representative maps of NLs in January and July. The findings of this study are important for policymakers and marine resource managers as the generated maps will provide valuable insights into the spatial and temporal distribution of ship noise in the region. The underwater noise level threshold that is harmful for marine organisms has not been agreed upon yet because it depends on various factors such as the frequency of noise and the type of marine life; however, numerous studies have shed light on its potential detrimental effects.

The results of the study provide insights into the distribution and intensity of ship noise in the Red Sea, focusing on the factors that influence its variability across different regions. One of the key findings is that the northern Red Sea experiences the highest ship NLs, particularly in areas close to the Suez Canal. The bathymetric characteristics of the Red Sea also play a crucial role in shaping the distribution of ship noise. The Red Sea has extended shallow regions along its coast; therefore, NLs are significantly attenuated in these near-surface depths. However, NLs increase with depth, reaching higher values in shallow and intermediate depths, and decrease as the depth increases. This complex pattern can be attributed to the interplay between factors such as temperature, pressure, and the bathymetric features of the region.

The noise maps reveal an interesting division of the Red Sea into eastern and western parts, distinguished by a peak in NL along its axis. This peak is directly associated with the main shipping lanes. Areas with high ship traffic, such as the coastal regions of Jeddah and Port Sudan, exhibit elevated NLs due to the continuous flow of vessels in these regions. In contrast, the southern Red Sea (SRS) regions, including areas such as Dahlak and Farasan, experience lower NLs because of the presence of islands and coral reefs that create shadows and reduce noise propagation near the coastal regions. Additionally, the subsequent restriction of shipping lanes to a narrow corridor in these areas contributes to the lower NLs in the SRS. However, a noteworthy exception to this trend is observed in the Suez region. Despite its shallow depths, NLs are notably high in this narrow Gulf. The sound sources, from the concentrated ship traffic passing through the Suez Canal region, produces the elevated NLs in this area.

Seasonal variations in ship noise propagation were also evident in the study, with higher NLs observed during winter compared to summer. These fluctuations may be influenced by factors such as weather conditions and changes in shipping activity as well as sound speed, mainly near the surface.

The findings of this study have significant implications for marine conservation and management efforts in the Red Sea. The identification of areas with high noise levels and an understanding of the factors influencing their variation provide a foundation for developing effective mitigation strategies. By implementing measures to minimize ship noise, such as regulating vessel routes and promoting the adoption of quieter propulsion systems, the negative impacts of noise pollution on marine species that heavily rely on sound for communication, navigation, and feeding can be mitigated. In addition, the interconnection between species in the Red Sea^53^ implies that future connectivity studies would greatly benefit from the incorporation of underwater sounds. By incorporating acoustic data into such investigations, the complex interactions and communication mechanisms among Red Sea organisms can be comprehensively elucidated.

However, significant knowledge gaps remain in the accuracy and validity of data used in our underwater modeling. Specifically, there is a scarcity of detailed data on the geoacoustic parameters of the deep layers of the Red Sea. The complex seafloor topography and variability of the ocean environment can also pose challenges in accurately modeling acoustic propagation. A ship’s propellers, hull, and other machinery further contribute to its sound signature. Additionally, the type and frequency of the sound emitted by a ship may vary depending on its operational conditions such as the vessel’s speed and water conditions. These limitations can result in approximations and constrain the models’ ability accurately simulate acoustic propagation in the region. In addition, the need for noise measurements and further calibration of the model should be underscored.

Despite these inherited uncertainties, this study is the first to investigate NLs in the Red Sea. While there may be some margin of uncertainty in terms of decibels, the results of this study serve as an essential starting point for further discussion on this topic and as a benchmark for future research. It underscores the need for more detailed investigations in specific regions, such as port areas and those close to the Suez Canal, where NLs are the highest. Moreover, it focuses on seafloor topography and related factors in terms of the physical characteristics of the ocean floor, such as the depth, contours, and composition. This work provides an invaluable overview of the coral reef areas at risk due to underwater noise, guiding ocean sampling efforts and conservation strategies. This information is essential for protecting the delicate ecosystems of the Red Sea and ensuring the long-term health of its marine biodiversity.

### Supplementary Information

Supplementary Information.

## Data Availability

Data is available on request from the authors. The data that support the findings of this study are available from the corresponding author (I.H), upon reasonable request.
