# Supplementary material for: Numerical investigation of shipping noise in the Red Sea
Source: Sci Rep. 2024 Mar 11;14:5851. doi: 10.1038/s41598-024-56523-2 (PMC10925598; doi:10.1038/s41598-024-56523-2)
Supplement: Supplementary file 1 — Supplementary Information. [file 41598_2024_56523_MOESM1_ESM.pdf]

Numerical Investigation of Shipping Noise in the Red Sea: Supplementary Information

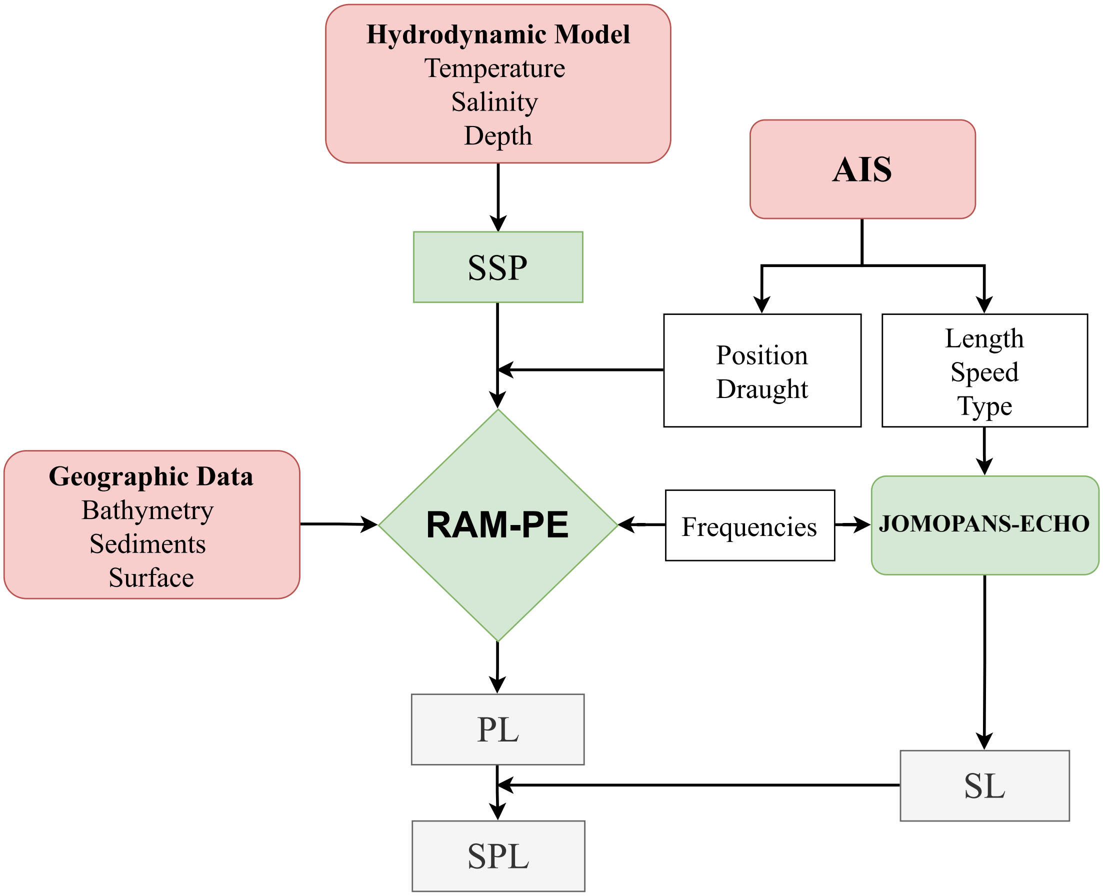

Fig. S1. Flow chart of the modeling process.

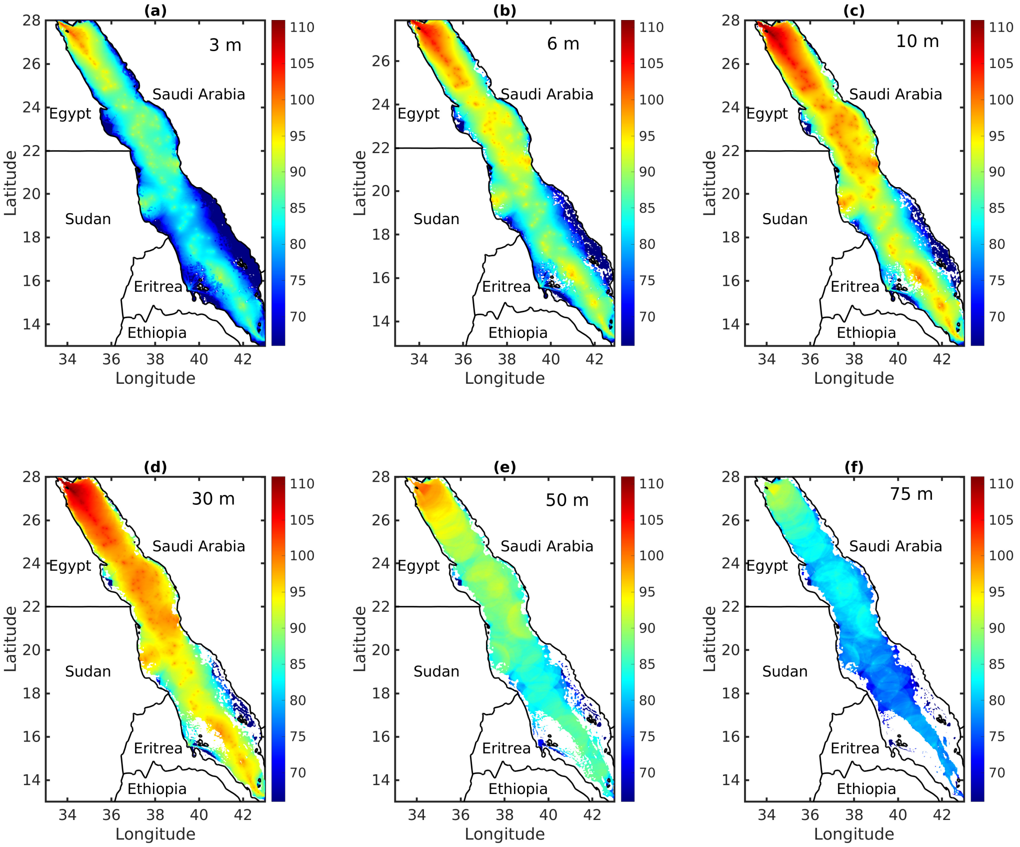

Fig. S2. Predicted spectral noise level ($dB\ re\ 1\mu Pa^2/Hz$) distribution for the 63 Hz decidecade band, represented by colors, on 11 January 2021 in the Red Sea at depths of (a) 3 m, (b) 6 m, (c) 10 m, (d) 30 m, (e) 50 m, and (f) 75 m. Map produced using Matlab R2023a (https://www.mathworks.com)
